# Using the Mooney Space to Characterize the Non-Affine Behavior of Elastomers

**DOI:** 10.3390/ma17051098

**Published:** 2024-02-28

**Authors:** Laura Moreno-Corrales, Miguel Ángel Sanz-Gómez, José María Benítez, Luis Saucedo-Mora, Francisco J. Montáns

**Affiliations:** 1 ETS de Ingeniería Aeronáutica y del Espacio, Universidad Politécnica de Madrid, Pza Cardenal Cisneros 3, 28040 Madrid, Spain; laura.mcorrales@upm.es (L.M.-C.); miguelangel.sanz@upm.es (M.Á.S.-G.); josemaria.benitez@upm.es (J.M.B.); luis.saucedo@upm.es (L.S.-M.); 2 Department of Materials, University of Oxford, Parks Road, Oxford OX1 3PJ, UK; 3 Department of Nuclear Science and Engineering, Massachusetts Institute of Technology, Cambridge, MA 02139, USA; 4 Department of Mechanical and Aerospace Engineering, Herbert Wertheim College of Engineering, University of Florida, Gainesville, FL 32611, USA

**Keywords:** rubber-like materials, hyperelasticity, polymers, elastomers, statistical theory, constitutive modeling

## Abstract

The formulation of the entropic statistical theory and the related neo-Hookean model has been a major advance in the modeling of rubber-like materials, but the failure to explain some experimental observations such as the slope in Mooney plots resulted in hundreds of micromechanical and phenomenological models. The origin of the difficulties, the reason for the apparent need for the second invariant, and the reason for the relative success of models based on the Valanis–Landel decomposition have been recently explained. From that insight, a new micro–macro chain stretch connection using the stretch tensor (instead of the right Cauchy–Green deformation tensor) has been proposed and supported both theoretically and from experimental data. A simple three-parameter model using this connection has been suggested. The purpose of this work is to provide further insight into the model, to provide an analytical expression for the Gaussian contribution, and to provide a simple procedure to obtain the parameters from a tensile test using the Mooney space or the Mooney–Rivlin constants. From different papers, a wide variety of experimental tests on different materials and loading conditions have been selected to demonstrate that the simple model calibrated only from a tensile test provides accurate predictions for a wide variety of elastomers under different deformation levels and multiaxial patterns.

## 1. Introduction

Because of its practical importance, understanding and modeling the nonlinear behavior of elastomers has been a major research in chemistry, materials, and continuum mechanics for a century. A major step toward this goal has been the introduction of the entropic statistical theory of polymers, which explained the nature of the nonlinear behavior and the shape of the stress–strain curve [1,2,3,4].

However, for more than 75 years, the failure of the statistical theory to explain some aspects of the observed behavior, such as the experimentally observed slope in the Mooney plots [5,6,7], has been disappointing [8], and hundreds of physics-based and phenomenological models have been proposed to overcome the limitations; in particular, the second invariant has been incorporated [6,7]. However, despite some improvements, problems remained and have been manifested by conflicting claims and unsolved issues [9]. Some of them have been: (1) the need for more than one test to characterize an isotropic incompressible material when only one modulus (one test) is needed to define the linear material [10]; (2) the need for the introduction of a second invariant or chain transverse (or tube) constraints [11]; (3) the failure of the full network model, i.e., sphere integration of the chain behavior to obtain the continuum one [12,13]; (4) the need for the modification of the chain stretch(es) (longitudinal and transverse) by averaging in the sphere [11,14]; (5) and a conceptual contradiction of affine deformations with the statistical theory [15,16]. The lack of sufficient understanding and the difficulty in selecting the appropriate model resulted in tens of papers comparing the predictive power of different models when parameters are characterized by a multitude of approaches. Some well-known comparative studies are [10,12,14,17,18,19,20,21,22,23].

Based on the consistency of the 3D extension of the statistical theory [9], and on some insights obtained from machine learning [24], a new micro–macro connection for the chain stretch has been proposed, where the stretch tensor replaces the Cauchy–Green deformation tensor from the original affine theory [4]. This results in an orientationally non-affine chain stretch, but which is consistent with neglecting the entropy changes from the network reorientation, as usually assumed, where only chain entropies are considered. It has been demonstrated that the new micro–macro relation solves and explains many standing issues like the slope in the Mooney plots [9]. The resulting model is also characterized from a (any) single stress–strain curve and results in accurate 3D predictions [9,24]. However, as also therein mentioned, it is expected that the network entropy changes when chains approach locking, so a more orientationally affine behavior is expected. A simple three-parameter full network model has been proposed recently under these considerations [25].

The purpose of this paper is to provide further insights into the model. There are four main contributions. (1) A closed form, simple, analytical expression for the model for moderately large stretches (within the Gaussian zone) is given. This closed form is important in developing many analytical studies and derivations. It is often considered an asset for many models, as it is for the Neo-Hookean model. (2) A detailed comparison of the present model with the Neo-Hookean model is performed, demonstrating the relevance of the orientationally non-affine deformations assumption in reproducing the experimental observations for general multiaxial loadings with parameters obtained from a single test. (3) It is demonstrated how the parameters of the model may be easily obtained from the Mooney space, or alternatively from the Mooney–Rivling constants, revealing also the importance of the lowest range of large stretches. (4) The model is verified against a large variety of experimental results for different elastomers. These data include true biaxial tests with different stretch ratios, different treatments, and different stretch levels. In all the predictions using the model, the three material parameters have been ontained only from a tensile test.

## 2. The Orientationally Non-Affine Chain Stretch

As above explained, the initial success of the statistical theory and the Neo-Hookean model by Wall [4]—who first noted that it entailed a Hookean behavior in shear—in explaining the shape of the stress–strain uniaxial relation was not followed by a satisfactory extension to 3D. Many researchers, starting from Mooney [5] and followed by Rivlin and co-workers [6,7,10,26,27,28,29,30], highlighted the failure of a theory based only on the first invariant $I_{1}^{C}$ of the Cauchy–Green deformation tensor, so they phenomenologically proposed the incorporation of an additional term. The Neo-Hookean model results in a constant in the Mooney $y-(1/\lambda_{u})$ plot

(1) $$y\left(1/\lambda_{u}\right)\equiv \frac{P_{u}\left(\lambda_{u}\right)}{2\left(\lambda_{u}-\frac{1}{\lambda_{u}^{2}}\right)}=C_{1}$$

where $\lambda_{u}$ is the uniaxial stretch and $P_{u}$ is the nominal stress. However, $y(1/\lambda_{u})$ is not constant in experiments but has a slope in the order of 1/10 —depending on the polymer; polyurethane elastomers may have slopes of the order of $C_{1}$ or even 10 times $C_{1}$ [31]. Mooney’s solution has been to incorporate such slope by adding a $C_{2}$ term

(2) $$y\left(1/\lambda_{u}\right)\equiv \frac{P_{u}}{2\left(\lambda_{u}-\frac{1}{\lambda_{u}^{2}}\right)}=C_{1}+C_{2}\left(\frac{1}{\lambda_{u}}\right)$$

using the Rivlin’s Cauchy–Green tensor invariants $I_{1}^{C}$ , $I_{2}^{C}$ , the strain energy is

(3) $$\Psi \left(I_{1},I_{2}\right)=C_{1}\left(I_{1}^{C}-3\right)+C_{2}\left(I_{2}^{C}-3\right)$$

However, Rivlin [6,7] also noted that the parameters $C_{1},C_{2}$ could not be considered as constants but were functions of the invariants themselves, i.e., $C_{1}\left(I_{1}^{C},I_{2}^{C}\right)$ , $C_{2}\left(I_{1}^{C},I_{2}^{C}\right)$ . $I_{i}^{C}$ represents the invariants of the right Cauchy–Green deformation tensor $C=F^{T}F=U^{2}$ , F is the deformation gradient, and U is the right stretch tensor. Noteworthy, the Neo-Hookean model is just the affine full integration in the sphere Ω of the chain function

(4) $$\psi_{ch}^{\mathrm{NH}}=\frac{1}{2}\left(3\mu^{\mathrm{NH}}\right)\left[{\left(\lambda_{ch}^{C}\right)}^{2}-1\right]\;\Rightarrow \;\Psi^{\mathrm{NH}}\left(C\right)=\int_{\Omega }\psi_{ch}^{\mathrm{NH}}\left(\lambda_{ch}^{C}\right)\frac{d\Omega }{\Omega }=\frac{1}{2}\mu^{\mathrm{NH}}\left(I_{1}^{C}-3\right)$$

where $\lambda_{ch}^{C}=\sqrt{r\cdot C\cdot r}$ is the chain stretch obtained from the right Cauchy–Green deformation tensor C and the chain direction in the reference configuration r , and $\mu^{\mathrm{NH}}=2C_{1}$ is the classical Neo-Hookean shear modulus. A corrected “Neo-Hookean” model using $\lambda_{ch}=r\cdot U\cdot r\neq \sqrt{r\cdot C\cdot r}$ , is

(5) $$\psi_{ch}=\frac{1}{2}\left(3\mu \right)\left(\lambda_{ch}^{2}-1\right)\;\Rightarrow \;\Psi \left(U\right)=\int_{\Omega }\psi_{ch}\left(\lambda_{ch}\right)\frac{d\Omega }{\Omega }$$

where μ is a shear-like modulus—see below for the correspondence with the classical $\mu^{\mathrm{NH}}$ . This model provides much more accurate results and, importantly, the correct 3D tendencies, including the observed slope in Mooney plots [9]. Model (5) is also physically consistent with the neglected entropy terms regarding the reorientation of the chains. Building upon this model, a new three-parameter model that incorporates two experimentally observed effects has been proposed: (1) a constant term to account for internal energy effects at low deformation levels [8,32] and (2) a chain-locking behavior which incorporates an increasingly orientationally affine deformation (assuming that chains near locking deform under more affine conditions). In the remaining part of the paper, important insights into the model, the Mooney representation of the model, and its predictive power for different elastomers are given.

## 3. Non-Affine Model with Three Parameters

**Figure 1:**
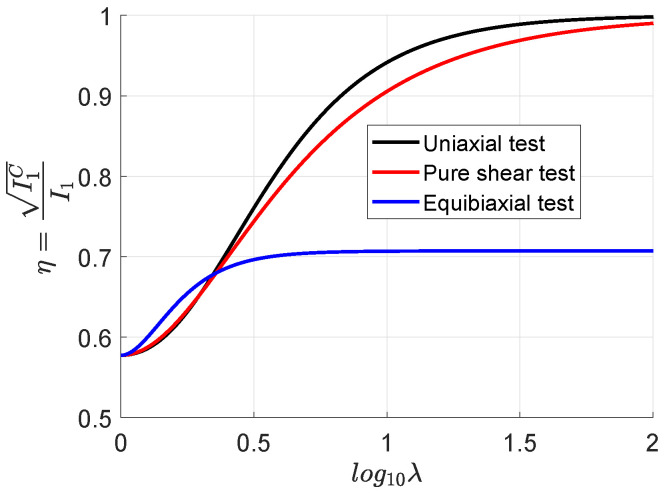
Evolution of the affinity parameter $\eta =\sqrt{I_{1}\left(C\right)}/I_{1}\left(U\right)=\sqrt{I_{1}^{C}}/I_{1}$ with the (logarithm of the) loading stretch for the uniaxial, pure shear and equibiaxial tests. Initially, all tests have $\eta =1/\sqrt{3}$ (affine and non-affine distributions are coincident), but at large stretches, the loading patterns mark a difference between affine and non-affine invariants. The equibiaxial loading case results in larger transverse constraints than uniaxial or pure shear ones. These constraints are more relevant near locking, and the parameter η accounts for such effects in general 3D cases.

Using Langevin distributions [8], where $L^{-1}\left(•\right)$ is the inverse Langevin function, the derivative of the chain energy $\psi_{ch}$ with respect to the chain stretch $\lambda_{ch}$ can be written as:

(6) $$\begin{array}{cc} \frac{d\psi_{ch}}{d\lambda_{ch}}=:P_{ch} & ≃P_{0}+\mu \eta \lambda^{lock}L^{-1}\left(\frac{{\tilde{\lambda }}_{ch}}{\eta \lambda^{lock}}\right) \end{array}$$

(7) $$\begin{array}{c} \approx P_{0}+3\mu \lambda_{ch}+\mathrm{nonlinear}\;\mathrm{high}\;\mathrm{order}\;\mathrm{terms} \end{array}$$

where ${\tilde{\lambda }}_{ch}$ is the effective chain stretch—see below—and $\eta \lambda^{lock}$ is the chain locking. The variable η is the conversion factor from the observed continuum uniaxial referential locking stretch $\lambda_{u}^{lock}$ to the chain locking, computed as—see motivation in [25]

(8) $$\eta =\frac{\sqrt{I_{1}\left(C\right)}}{I_{1}\left(U\right)}$$

where $I_{1}\left(•\right)$ stands for the first invariant of (•) ; see Figure 1. At very large stretches, the following values are obtained.

$$\left\{\begin{array}{c} \eta \approx 1\;\;\;\mathrm{for}\;\mathrm{uniaxial}\;\mathrm{loading}\;\mathrm{patterns}\\ \eta \approx 1\;\;\;\mathrm{for}\;\mathrm{pure}\;\mathrm{shear}\;\mathrm{loading}\;(\mathrm{but}\;\mathrm{smaller}\;\mathrm{than}\;\mathrm{for}\;\mathrm{uniaxial}\;\mathrm{with}\;\mathrm{the}\;\mathrm{same}\;\mathrm{stretch})\\ \eta \approx 1/\sqrt{2}\;\;\;\mathrm{for}\;\mathrm{equibiaxial}\;\mathrm{patterns} \end{array}\right.$$

These values are consistent with the approximate relations between locking stretches for those types of experiments. An estimation of the reference locking stretch for the chain is $\lambda^{lock}=\lambda_{u}^{lock}/\eta \left(\lambda_{u}^{lock}\right)$ , where $\lambda_{u}^{lock}$ is the macroscopic locking stretch obtained during a tensile test, and $\eta \left(\lambda_{u}^{lock}\right)$ is the value of the η function at that stretch $\lambda_{u}^{lock}$ for the uniaxial test. Equation (7) has two addends. The second addend corresponds to the classical statistical (Langevin) theory [8,18,33] with the exception of the presence of the loading mode factor η accounting for chain constraints. The first addend in the chain tension $P_{ch}$ —term $P_{0}$ in Equation (7)—corresponds to an internal energy contribution. This contribution can be considered approximately constant, and it is relevant only for relatively small stretches (e.g., about 50–100%), see [8,32] p. 32, but dominates the tension near the infinitesimal range—below 10% of stretches. It is noteworthy that this constant term at small strains has also been obtained in the data-driven determination of the chain function from experiments; see Ref. [24]. Furthermore, $P_{0}$ can take negative values and that still $P_{ch}\left(\lambda_{ch}=1\right)>0$ . Hence, even when this term is neglected at large deformations, it cannot be neglected when determining the constants if the shear modulus is obtained in that regime.

Several works have used the affine and non-affine behavior of polymer chains to characterize the transition between the microscopic constitutive model and the continuous solid [11,25,34,35], but in most of them, the consideration of non-affinity does not refer to the non-affinity in the orientation of the chains, but rather refers to the amount of the effective stretch in a given chain direction with respect to the continuum one. For example, the continuum deformation tensor to compute the chain stretch is typically the Cauchy–Green (quadratic) deformation tensor. The non-affine stretch $\lambda_{ch}$ is computed herein from the continuum stretch tensor U and the chain direction r (which is treated as a spatial direction, not a specific chain direction) as

(9) $$\begin{array}{cc} \lambda_{ch} & =r⊗r:U=\sum_{i=1}^{3}\lambda_{i}r_{i}^{2}={\left[r^{2}\right]}^{T}\left[\lambda \right]\\ \; & =\left[\begin{array}{ccc} \cos^{2}\phi \sin^{2}\theta & \sin^{2}\phi \sin^{2}\theta & \cos^{2}\theta \end{array}\right]\left[\begin{array}{c} \lambda_{1}\\ \lambda_{2}\\ \lambda_{3} \end{array}\right]\\ \; & =\lambda_{3}\cos^{2}\theta +\lambda_{1}\cos^{2}\phi \sin^{2}\theta +\lambda_{2}\sin^{2}\phi \sin^{2}\theta \end{array}$$

in this expression, $\lambda_{i}$ are the principal continuum stretches and ϕ,θ are, respectively, the azimuthal and polar spherical angles of the chain with respect to the principal directions. The microstretch $\lambda_{ch}$ is the one consistent with biaxial experimental data at moderately large stretches; see [24]. However, near locking, it is to be expected that chains reorient statistically toward the stretched directions because locking behavior seems experimentally more consistent with the affine assumption. Unfortunately, there is still no experimentally verified theory which incorporates the network reorientation in the entropy, so the increasing relevance of that term with very large deformations results in the effective average reorientation of chains. Then, this effect is incorporated phenomenologically by considering an average effective reoriented chain with stretch— p=1 corresponds to the orientationally non-affine case, verified experimentally up to moderate stretches, and p=2 corresponds to the limit affine case expected at chain locking.

(10) $${\tilde{\lambda }}_{ch}=\sqrt[{p}]{r⊗r:U^{p}}=\sum_{i=1}^{3}\sqrt[{p}]{\lambda_{i}^{p}r_{i}^{2}}\;\;\mathrm{with}\;\;p\left(\lambda_{ch}\right)=\frac{2+\exp\left(-2\lambda_{ch}+\lambda^{lock}\right)}{1+\exp\left(-2\lambda_{ch}+\lambda^{lock}\right)}\in \left[1,2\right)$$

The parameters $P_{0}$ , μ and $\lambda^{lock}$ are the material fitting parameters with a clear physical interpretation. For deformations sufficiently small, the locking effect can be neglected (typically 30% of the locking stretch), which is known as the Gaussian distribution case, e.g., [35,36].

The model considers a full network of chains isotropically oriented, so a chain oriented in a given direction represents all chains oriented in that direction. Then, the derivative of the continuum stored energy $\Psi \left(U\right)=\int_{\Omega }\psi_{ch}\left(\lambda_{ch}\right)d\Omega /\Omega$ is computed from the chain rule as

(11) $$\begin{array}{cc} \frac{\partial \Psi (\lambda_{1},\lambda_{2},\lambda_{3})}{\partial \lambda_{i}}\; & =\int_{\Omega }\frac{d\psi_{ch}}{d\lambda_{ch}}\frac{d\lambda_{ch}}{d\lambda_{i}}\frac{d\Omega }{\Omega }\\ \; & =\int_{0}^{2\pi }\int_{0}^{\pi }\frac{d\psi_{ch}}{d\lambda_{ch}}\frac{d\lambda_{ch}}{d\lambda_{i}}\frac{\sin\theta d\theta d\phi }{4\pi }\\ \; & =\int_{0}^{2\pi }\int_{0}^{\pi }\left[P_{0}+\mu \eta \lambda^{lock}L^{-1}\left(\frac{{\tilde{\lambda }}_{ch}}{\eta \lambda^{lock}}\right)\right]\frac{\partial {\tilde{\lambda }}_{ch}}{\partial \lambda_{i}}\frac{\sin\theta d\theta d\phi }{4\pi }\\ \; & =\sum_{k=1}^{n_{p}}\left[P_{0}+\mu \eta \lambda^{lock}L^{-1}\left(\rho_{\left(k\right)}\right)\right]\frac{\partial {\tilde{\lambda }}_{ch}}{\partial \lambda_{i}}w_{k} \end{array}$$

where the last line is the numerical integration of $n_{p}$ points of quadrature, with $w_{k}$ being the weights of integration (such that $\sum w_{k}=1$ ). In our case, we use the quadrature points proposed by Bazant and Oh [37] with $n_{q}=42$ , which is the same one used by Miehe et al. in their non-affine model [11]. The non-affine stretch is

(12) $${\tilde{\lambda }}_{ch\left(k\right)}:=\sum_{j=1}^{3}\sqrt[{p}]{\lambda_{j}^{p}r_{j(k)}^{2}},\;\;\mathrm{and}\;\rho_{\left(k\right)}=\frac{\sum_{j=1}^{3}\sqrt[{p}]{\lambda_{j}^{p}r_{j(k)}^{2}}}{\eta \lambda^{lock}}\;\;\;$$

where

(13) $$\left[\begin{array}{c} r_{1(k)}^{2}\\ r_{2(k)}^{2}\\ r_{3(k)}^{2} \end{array}\right]=\left[\begin{array}{c} \cos^{2}\phi_{\left(k\right)}\sin^{2}\theta_{\left(k\right)}\\ \sin^{2}\phi_{\left(k\right)}\sin^{2}\theta_{\left(k\right)}\\ \cos^{2}\theta_{\left(k\right)} \end{array}\right]=:r_{\left(k\right)}^{2}$$

A main problem of Langevin statistical models is the evaluation of the inverse of the Langevin function. There is no analytical expression for that inverse function. Furthermore, it is difficult to accurately evaluate the inverse Langevin function because of the asymptotic behavior near locking. Thus, some studies are dedicated to this issue [38,39,40,41,42,43,44]. However, in the present case, it is relevant to separate the Gaussian linear zone from the nonlinear locking one. The Petrosyan [45] approximation to the inverse Langevin function (with a maximum error of 0.18% ) conveniently splits the linear (Gaussian) and nonlinear parts

(14) $$L^{-1}\left(\rho \right)=\underset{\mathrm{linear}}{\underset{︸}{3\rho }}+\underset{\mathrm{nonlinear}}{\underset{︸}{\frac{\rho^{2}}{5}\sin\left(\frac{7\rho }{2}\right)+\frac{\rho^{3}}{1-\rho }}}$$

the nonlinear contribution is

(15) $$N\left(\rho \right):=L^{-1}\left(\rho \right)-3\rho =\frac{\rho^{2}}{5}\sin\left(\frac{7\rho }{2}\right)+\frac{\rho^{3}}{1-\rho }$$

then, the approximation symbol is used because of the consideration of $\lambda_{ch}$ in the linear part and ${\tilde{\lambda }}_{ch}$ in the nonlinear one and $\partial \lambda_{ch}/\partial \lambda_{i}=r_{i(k)}^{2}$

(16) $$\begin{array}{cc} \frac{\partial \Psi (\lambda_{1},\lambda_{2},\lambda_{3})}{\partial \lambda_{i}}\; & ≃\frac{\partial \Psi^{L}}{\partial \lambda_{i}}+\frac{\partial \Psi^{NL}}{\partial \lambda_{i}}=\frac{1}{3}P_{0}+\frac{\mu }{5}\left[2\lambda_{i}+\left(\lambda_{1}+\lambda_{2}+\lambda_{3}\right)\right]\\ \; & +\sum_{k=1}^{n_{p}}\mu \eta \lambda^{lock}w_{k}N\left(\rho_{\left(k\right)}\right)\frac{\partial {\tilde{\lambda }}_{ch\left(k\right)}}{\partial \lambda_{i}} \end{array}$$

where the second line is the non-Gaussian contribution [46,47], and with

(17) $$\rho_{\left(k\right)}=\frac{{\tilde{\lambda }}_{ch\left(k\right)}}{\eta \lambda^{lock}}=\frac{\sum_{i=1}^{3}\sqrt[{p}]{\lambda_{j}^{p}r_{j(k)}^{2}}}{\eta \lambda^{lock}}$$

the derivative $\partial {\tilde{\lambda }}_{ch}/\partial \lambda_{i}$ is

(18) $$\frac{\partial {\tilde{\lambda }}_{ch\left(k\right)}}{\partial \lambda_{i}}={\left(\frac{\lambda_{i}}{{\tilde{\lambda }}_{ch}}\right)}^{p-1}r_{i\left(k\right)}^{2}+\frac{1}{p}\frac{dp}{d\lambda_{ch}}r_{i\left(k\right)}^{2}\left[\frac{\sum_{m=1}^{3}\lambda_{m}^{p}r_{m\left(k\right)}^{2}\ln\lambda_{m}}{{\tilde{\lambda }}_{ch\left(k\right)}^{p-1}}-{\tilde{\lambda }}_{ch\left(k\right)}\ln{\tilde{\lambda }}_{ch\left(k\right)}\right]$$

It is important to remark here that in contrast to the formulation in [25], the Gaussian case is integrated exactly, and only the non-Gaussian contribution needs to be integrated numerically. This is relevant because Mooney plots are only relevant in the Gaussian zone. If $\bar{p}$ denotes the pressure-like Lagrange multiplier of the incompressible case, I is the identity tensor, and A denotes the Green–Lagrange strain tensor, while the incompressible case gives the following second Piola–Kirchhoff and Piola stress tensors, respectively, S and P :

(19) $$S=\bar{p}C^{-1}+\frac{d\Psi }{dA}\;\mathrm{and}\;P=FS=\bar{p}F^{-T}+F\frac{d\Psi (A)}{dA}$$

with

(20) $$\frac{d\Psi (A)}{dA}=\sum_{i=1}^{3}\frac{1}{\lambda_{i}}\frac{d\Psi (\lambda_{i})}{d\lambda_{i}}N_{i}⊗N_{i}=\left[\frac{1}{3}P_{0}+\frac{1}{5}\mu \left(U:I\right)\right]U^{-1}+\frac{2}{5}\mu I$$

where $N_{i}$ represents the eigenvectors of U . i.e., the Piola stress is

(21) $$\begin{array}{cc} P & =FS=\bar{p}F^{-T}+\left[\frac{1}{3}P_{0}+\frac{1}{5}\mu \left(U:I\right)\right]R+\frac{2}{5}\mu F\\ \; & =R\left\{\bar{p}U^{-1}+\left[\frac{1}{3}P_{0}+\frac{1}{5}\mu \left(U:I\right)\right]I+\frac{2}{5}\mu U\right\} \end{array}$$

where $R=FU^{-1}$ is the rotation from the right polar decomposition of F .

In the typical quasi-incompressible case, the stored energy can be written as $W(J,\lambda_{1}^{d},\lambda_{2}^{d}$ $,\lambda_{3}^{d})=U\left(J\right)+\Psi \left(\lambda_{1}^{d},\lambda_{2}^{d},\lambda_{3}^{d}\right)$ with $J=\lambda_{1}\lambda_{2}\lambda_{3}$ being the determinant of the deformation gradient tensor F and $\lambda_{i}^{d}=J^{-1/3}\lambda_{i}$ being the isochoric stretches. Taking into account that

(22) $$\frac{\partial \lambda_{j}^{d}}{\partial \lambda_{i}}=J^{-1/3}\left(\delta_{ij}-\frac{1}{3}\frac{\lambda_{j}^{d}}{\lambda_{i}^{d}}\right)\;\mathrm{and}\;\frac{\partial J}{\partial \lambda_{i}}=\frac{J}{\lambda_{i}}$$

it is obtained

(23) $$\begin{array}{cc} \frac{\partial W(J,\lambda_{1}^{d},\lambda_{2}^{d},\lambda_{3}^{d})}{\partial \lambda_{i}} & =\frac{\partial U(J)}{\partial \lambda_{i}}+\frac{\partial \Psi (\lambda_{1}^{d},\lambda_{2}^{d},\lambda_{3}^{d})}{\partial \lambda_{i}}\\ \; & =U^{\prime }\left(J\right)\frac{\partial J}{\partial \lambda_{i}}+\sum_{j=1}^{3}\frac{\partial \Psi (\lambda_{1}^{d},\lambda_{2}^{d},\lambda_{3}^{d})}{\partial \lambda_{j}^{d}}\frac{\partial \lambda_{j}^{d}}{\partial \lambda_{i}}\\ \; & =\frac{J}{\lambda_{i}}U^{\prime }+J^{-1/3}\sum_{j=1}^{3}\frac{\partial \Psi }{\partial \lambda_{j}^{d}}\left(\delta_{ij}-\frac{1}{3}\frac{\lambda_{j}^{d}}{\lambda_{i}^{d}}\right) \end{array}$$

where $\partial \Psi /\partial \lambda_{j}^{d}$ is given in Equation (11) by replacing $\lambda_{i}$ by $\lambda_{i}^{d}$ , and U(J) depends on the choice for the penalty function. For the Gaussian case

(24) $$\frac{\partial W}{\partial \lambda_{i}}=\frac{J}{\lambda_{i}}U^{\prime }\left(J\right)+\sum_{j=1}^{3}J^{-1/3}\left\{\frac{1}{3}P_{0}+\frac{\mu }{5}\left[2\lambda_{j}^{d}+\mathrm{tr}\left(J^{-1/3}U\right)\right]\right\}\left(\delta_{ij}-\frac{1}{3}\frac{\lambda_{j}^{d}}{\lambda_{i}^{d}}\right)$$

and

(25) $$S=\sum_{i=1}^{3}\frac{1}{\lambda_{i}}\frac{\partial W}{\partial \lambda_{i}}N_{i}⊗N_{i}\;\mathrm{and}\;P=FS=\sum_{i=1}^{3}\frac{\partial W}{\partial \lambda_{i}}n_{i}⊗N_{i}$$

where $n_{i}=RN_{i}$ are the eigenvectors of the left Cauchy–Green deformation tensor.

A relevant case is that of homogeneous deformation. For any given state, we can assume there are deformations in the principal axis. In most tests, one of the directions—label it as the third one—remains unloaded, so the stress state is biaxial, and the stretch in that axis is given by the incompressibility condition; namely $\lambda_{3}=1/\left(\lambda_{1}\lambda_{2}\right)$ . It is in the interest of simplifying analytical derivations in homogeneous tests to consider the incompressible case. In this case,

(26) $$P_{3}=0\Rightarrow \frac{1}{\lambda_{3}}p+\frac{\partial \Psi }{\partial \lambda_{3}}=0\Rightarrow p=-\lambda_{3}\frac{\partial \Psi (\lambda_{1},\lambda_{2},\lambda_{3})}{\partial \lambda_{3}}$$

and

(27) $$P_{i}=\frac{\partial \Psi }{\partial \lambda_{i}}-\frac{\lambda_{3}}{\lambda_{i}}\frac{\partial \Psi }{\partial \lambda_{3}}$$

For the Gaussian range of deformations, the explicit expression

(28) $$P_{\alpha }\equiv P_{\alpha }^{G}=\frac{1-\lambda_{3}/\lambda_{\alpha }}{15}\left[5P_{0}+3\mu \left(\lambda_{1}+\lambda_{2}+\lambda_{3}\right)+6\mu \left(\lambda_{\alpha }+\lambda_{3}\right)\right]\;\mathrm{with}\;\alpha =1,2$$

is obtained, whereas the non-Gaussian case gives the additional term

(29) $$P_{\alpha }=P_{\alpha }^{G}+\sum_{k=1}^{n_{p}}\left[\mu \eta \lambda^{lock}N\left(\rho_{\left(k\right)}\right)w_{k}\right]\left(r_{\alpha (k)}^{2}-\frac{\lambda_{3}}{\lambda_{i}}r_{3(k)}^{2}\right)$$

Predictions for the typical experiments are obtained using these formulae, employing

- Uniaxial test: $\lambda_{1}=\lambda_{u}$ (uniaxial stretch), and $\lambda_{2}=\lambda_{3}=1/\sqrt{\lambda_{1}}$ ;
- Equibiaxial test: $\lambda_{1}=\lambda_{2}=\lambda_{eq}$ (equibiaxial stretch), and $\lambda_{3}=1/\lambda_{eq}^{2}$ ;
- Pure shear: $\lambda_{1}=\lambda_{ps}$ (strip test stretch), and $\lambda_{2}=1,\;\lambda_{3}=1/\lambda_{ps}$ .

However, Equation (28) is valid for any test in which one axis—labeled as the third one—is unloaded. In incompressible cases, since the pressure comes from equilibrium, one axis may be taken as the zero reference.

In the case of uniaxial tests, it is typical to plot the experimental data, and hence the model fit, in the P−λ axes. The effective uniaxial modulus can be obtained by setting $\lambda_{u}=1+\varepsilon$ , where ε is the infinitesimal strain. In this case, the relevant Gaussian case gives

(30) $$\begin{array}{cc} P_{1} & =\frac{1-\frac{1}{\left(1+\varepsilon \right)\sqrt{1+\varepsilon }}}{15}\left[5P_{0}+3\mu \left(1+\varepsilon +2\frac{1}{\sqrt{1+\varepsilon }}\right)+6\mu \left(1+\varepsilon +\frac{1}{\sqrt{1+\varepsilon }}\right)\right]\\ \; & =\varepsilon \left(\frac{21}{10}\mu +\frac{1}{2}P_{0}\right)-\varepsilon^{2}\left(\frac{93}{40}\mu +\frac{5}{8}P_{0}\right)+\varepsilon^{3}\left(\frac{251}{80}\mu +\frac{35}{48}P_{0}\right)+\ldots \end{array}$$

to compare, the classical Neo-Hookean model gives

(31) $$\begin{array}{cc} P_{1} & =\mu^{NH}\lambda_{u}-\frac{1/\sqrt{\lambda_{u}}}{\lambda_{u}}\mu^{NH}\frac{1}{\sqrt{\lambda_{u}}}=\mu^{NH}\left(\lambda_{u}-\frac{1}{\lambda_{u}^{2}}\right) \end{array}$$

(32) $$\begin{array}{cc} \; & =3\mu^{NH}\varepsilon -3\mu^{NH}\varepsilon^{2}+4\mu^{NH}\varepsilon^{3}+\ldots \end{array}$$

The comparison of both models for infinitesimal strains $\varepsilon^{2}\to 0$ give the relation between the moduli of both models

(33) $${\left.\frac{dP_{1}}{d\lambda_{u}}\right|}_{\lambda_{u}=1}=3\mu^{NH}=\frac{21}{10}\mu +\frac{1}{2}P_{0}$$

this relation guarantees the same initial slope in the predictions by both models in a tensile test. Additionally, for a given stretch $\lambda_{u}$ , the slope for the tensile test is

(34) $$\frac{dP_{1}}{d\lambda_{u}}=\frac{1}{10\lambda_{u}^{3}}\left(16\mu +6\lambda_{u}^{3}\mu -\lambda_{u}^{3/2}\mu +5\sqrt{\lambda_{u}}P_{0}\right)$$

so for very large strains—recall that we are considering the Gaussian case

(35) $$\lim_{\lambda_{u}\to \infty }\frac{dP_{1}}{d\lambda_{u}}=\frac{3}{5}\mu$$

which is to be compared to the Neo-Hookean value $\mu^{\mathrm{NH}}$ —cf. Equation (31) for $\lambda_{u}\to \infty$

(36) $$\mu^{NH}=\frac{3}{5}\mu$$

Remarkably, $P_{0}$ affects the initial slope—Equation (33)—but not the behavior at large stretches—Equation (35). Note that for $P_{0}=0$ , Equation (33) gives $\mu^{\mathrm{NH}}=7/10\;\mu$ and Equation (36) gives $\mu^{\mathrm{NH}}=6/10\;\mu$ (again the μ/10 correction). In summary, from the initial slope and the intermediate slope (large moderate stretches, so the locking effect is not important), the two parameters of the model, namely μ and $P_{0}$ , can be determined, the former from Equation (35) and the latter with the computed μ and Equation (33).

## 4. Mooney Space Representation

Mooney’s plot is just another way of plotting the same tensile test experimental databut weighting visually the initial part of the experiment by using the representation in Equation (1). The Neo-Hookean model contradicts experimental evidence, where a slope in the order of 1/10 is observed [8,28]. This problem motivated Mooney’s phenomenological proposal of using a $C_{2}$ constant over $x=1/\lambda_{u}$ (or equivalently the $I_{2}^{C}$ invariant) which corrected the statistical theory to accommodate the experimental slope in that plot. The relevance of the $I_{2}^{C}$ invariant has been explained in many papers [8,48,49,50]. The slope in Mooney plots has also been the center of attention in fitting constitutive models [29,51,52,53]. In the herein proposed model, the Mooney slope is obtained naturally from the statistical theory. The model’s slope at $x=1/\lambda_{u}=1$ can be computed by considering the power series in δ ( δ<0 for a tensile test), where x=1+δ , so $\lambda_{u}=1/\left(1+\delta \right)$ . To this end, the Mooney plot function is

(37) $$\begin{array}{cc} y & =\frac{1}{30{\left(\frac{1}{x}\right)}^{3/2}+30}\left[12\mu +9\mu {\left(\frac{1}{x}\right)}^{3/2}+5P_{0}\sqrt{\frac{1}{x}}\right] \end{array}$$

whose expansion series in δ=x−1 is

(38) $$\begin{array}{c} y=\left(\frac{7}{20}\mu +\frac{1}{12}P_{0}\right)+\delta \left(\frac{3}{80}\mu +\frac{1}{48}P_{0}\right)-\delta^{2}\left(\frac{3}{160}\mu +\frac{1}{32}P_{0}\right)+\delta^{3}\left(\frac{7}{1280}\mu +\frac{17}{768}P_{0}\right)+\ldots \end{array}$$

For δ=0 , the previous expression of $\mu^{\mathrm{NH}}/2$ with $\mu^{\mathrm{NH}}$ given in Equation (36) is recovered. Then, the pursued slope is

(39) $$\frac{dy}{dx}=\frac{\frac{9}{x}\mu -5P_{0}+\frac{10}{x\sqrt{x}}P_{0}}{60x\sqrt{x}{\left(\frac{1}{x}\sqrt{\frac{1}{x}}+1\right)}^{2}}$$

whose expansion is

(40) $$\frac{dy}{dx}=\left(\frac{3}{80}\mu +\frac{1}{48}P_{0}\right)-\left(\frac{3}{80}\mu +\frac{1}{16}P_{0}\right)\delta +\left(\frac{21}{1280}\mu +\frac{17}{256}P_{0}\right)\delta^{2}+\ldots$$

now, at x=1 (δ=0) , the slope is

(41) $${\left.\frac{dy}{dx}\right|}_{x=\lambda_{u}=1}=\frac{3}{80}\mu +\frac{1}{48}P_{0}$$

then, Mooney plots may be used to identify the parameters of the model in a more simple way from the y -value at x=1 —call it $C_{1}$ , the Neo-Hookean constant, and the slope—call it $C_{2}$ , the Mooney constant. The solution is

(42) $$\mu =5C_{1}-20C_{2}\;\mathrm{and}\;P_{0}=84C_{2}-9C_{1}$$

If $P_{0}=0$ , as in the Neo-Hookean model, a nonvanishing initial slope $C_{2}=9/84C_{1}$ is still obtained, which is of the order of $C_{1}/10$ . The slope changes in general with deformation, but an almost constant slope is obtained for $P_{0}=-48/80\mu$ , and there is a vanishing initial one for $P_{0}\approx -2\mu$ . Of course, using Equations (37) and (39), the combination of function and slope values at any stretch, or two values at different stretches in the Gaussian zone, may be used to determine μ and $P_{0}$ by solving the linear system of equations.

## 5. Prediction of Different Sets of Experiments in Elastomers

In this section, predictions for different materials under a variety of loading conditions are given. Experimental data for several tests have been obtained from several sources in the literature. Material parameters have been obtained using the previous approach, extracting $P_{0}$ and μ from the estimated y-intercept at λ=1 and the overall slope in Mooney plots, and from the estimated locking stretch in P−λ , plots have been digitalized for this work. In several tests, we had to digitalize the stress–strain data from P−λ plots. Unfortunately, the errors in Mooney plots are magnified, which add to the more significative experimental errors at low stretches. Hence, sometimes, parameters have been refined from the adjustment to the resulting P−λ plot.

### 5.1. Prediction of the Treloar Tests [54]

**Figure 2:**
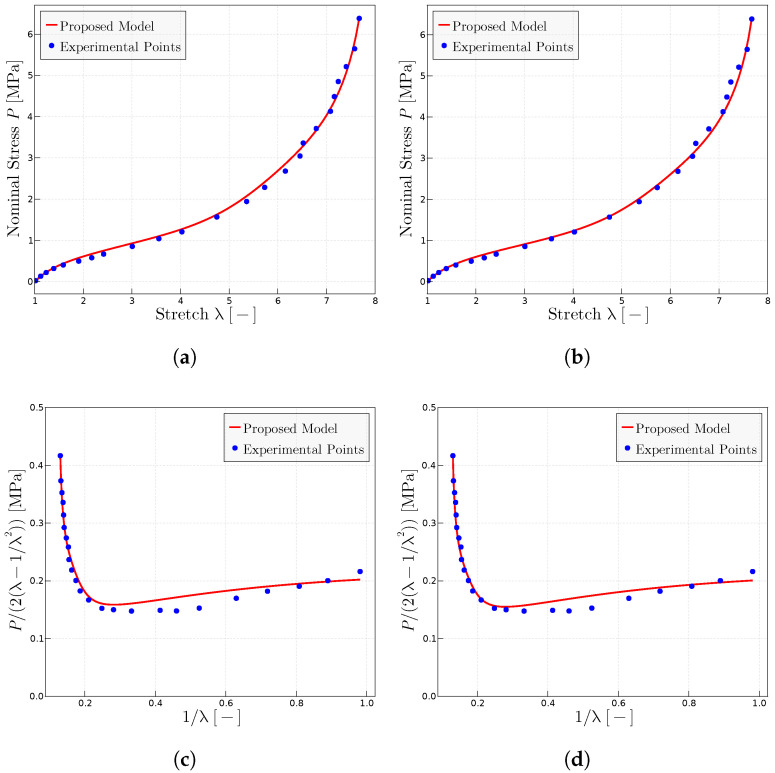
Predictions for the Treloar’s experimental data extracted from refs. [48,54]. (**a**) Treloar’s experiments in P−λ representation using the model proposed with the parameters $P_{0}=1.04$ MPa, μ=0.318 MPa and $\lambda_{u}^{\mathrm{lock}}=8.83$ . (**b**) Predictions in P−λ representation using the parameters $P_{0}=1.1$ MPa, μ=0.3 MPa and $\lambda_{u}^{\mathrm{lock}}=8.78$ . (**c**) Mooney space prediction with $P_{0}=1.04$ MPa, μ=0.318 MPa and $\lambda_{u}^{\mathrm{lock}}=8.83$ . (**d**) Mooney space prediction with $P_{0}=1.1$ MPa, μ=0.3 MPa and $\lambda_{u}^{\mathrm{lock}}=8.78$ .

Figure 2 shows the predictions for the Treloar tests [54] using different slightly different parameters. Figure 2a,b show the conventional P−λ representation, whereas Figure 2c,d show the Mooney plot representation. It is seen that the model naturally represents the Mooney slope. It is worth noting that the P−λ data and the Mooney data have been obtained from different sources, namely the former from [54] and the latter from [48]. It has been observed in this case that capturing accurately the Mooney slope resulted in worse predictions in the P−λ representation, so the given parameters are a trade-off manual adjustment between both representations.

### 5.2. Grumbell et al. Experiments on Different Natural Rubber Vulcanizates [28]

The Grumbell et al. experiments of Ref. [28] are predicted in this section.

In Ref. [28], the authors describe the samples and the conditions under which they performed the experiments. Furthermore, they also emphasize the importance of characterizing the behavior of rubber using both $C_{1}$ and $C_{2}$ constants (y-intercept and slope in Mooney plots) of the Mooney–Rivlin model.

**Figure 3:**
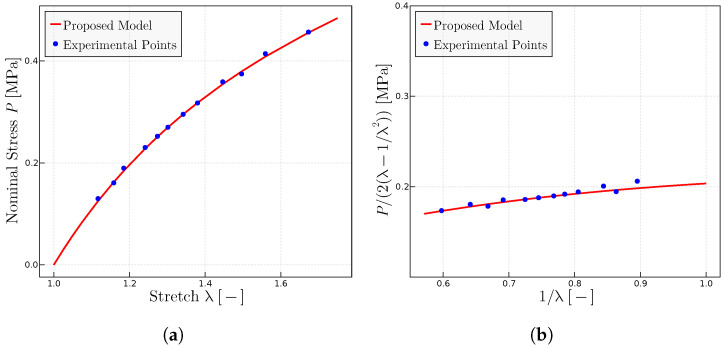
Experimental data extracted from Ref. [28]. (**a**) Uniaxial tests using the parameters $P_{0}$ = 1.925 MPa, μ = 0.115 MPa and $\lambda_{u}^{\mathrm{lock}}$ = 7.5. (**b**) Mooney plots with the same parameters.

**Figure 4:**
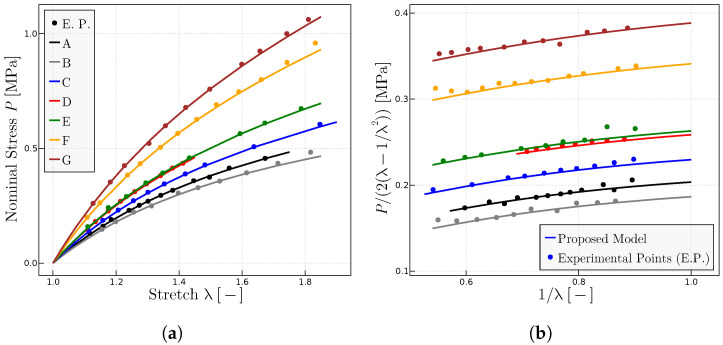
Predictions for different rubbers with experimental data given in Ref. [28] when $P_{0}$ is kept constant. (**a**) P−λ representation of a range of natural rubber vulcanizates. (**b**) Mooney representation for the same rubbers. Fitted model parameters are given in Table 1.

Regarding the prediction of the experiments in [28] using the proposed model, the rubber therein labeled A has been used as the reference rubber to select the initial parameters: in particular to estimate $P_{0}$ and $\lambda_{u}^{lock}$ , which are kept fixed for the other compounds. Only μ is adjusted to comply with the $C_{1}$ parameter (shear modulus or y-intercept) in the Mooney space. Since these experiments are not close to the locking zone, the predictions are quite insensitive to variations of $\lambda_{u}^{lock}$ . Figure 3 shows the obtained results for rubber A, whereas Figure 4 shows the results for all the compounds.

Table 1 summarizes the obtained parameters for the predictions of the experiments in Figure 4 for the varied tested range of natural rubber vulcanizates.

**Figure 5:**
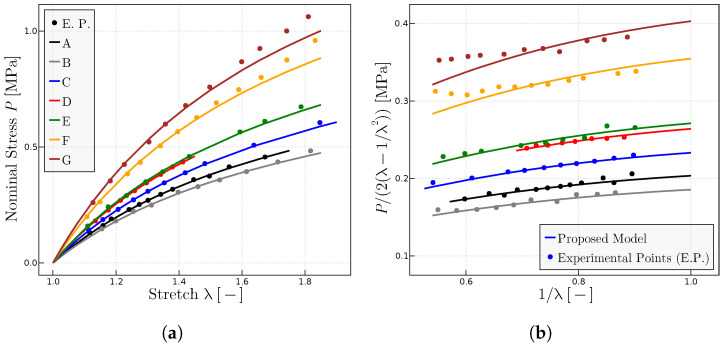
Predictions for different rubbers with experimental data given in Ref. [28] when μ is kept constant. (**a**) P−λ representation of a range of natural rubber vulcanizates. (**b**) Mooney representation for the same rubbers. The fitted model parameters are given in Table 2.

To analyze the influence of the parameter $P_{0}$ versus that of μ , Figure 5 shows the predictions when parameter μ is kept constant and $P_{0}$ is varied to adjust the experimental data. In practice, this implies a modification of the initial slope $m_{0}$ in the P−λ representation because $P_{0}=2\;\left(m_{0}-0.21\mu \right)$ while the Gaussian slope $m_{1}$ in the P−λ representation for large stretches is maintained as $m_{1}=\frac{3}{5}\;\mu$ for all rubbers. Table 2 summarizes the parameters for the predictions in Figure 5.

### 5.3. Mullins Experiments on Rubbers with Different Composition and Processing Conditions [27]

The proposed model is used to predict the behavior of dry rubbers that have been subjected to various experiments [27], as for example, rubbers that have been built with different peroxide concentrations, rubbers that have been processed with distinct periods of vulcanization, or rubbers whose initial molecular weights have been modified. The composition, processing and conditions during these experiments are detailed in Ref. [27]. The stretch range in these experiments is large enough to be significatively affected by the locking stretch, i.e., the typical upturn is clearly observed in the stress–strain curves, so in this case, the parameter $\lambda_{u}^{lock}$ is relevant in capturing that upturn.

**Figure 6:**
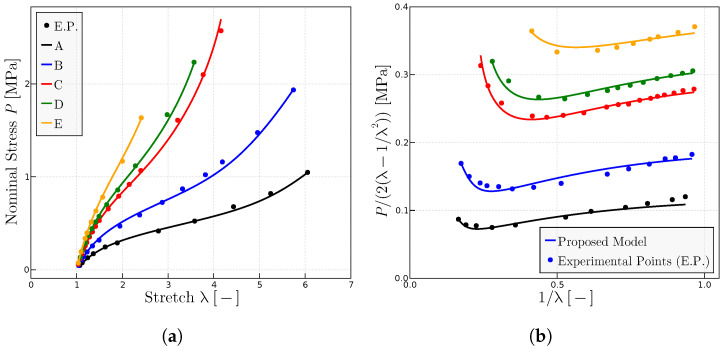
Predictions for different rubbers with experimental data given in Ref. [27]. (**a**) P−λ representation of different rubbers with different concentration of peroxide; parts of peroxide per 100 parts rubber of A (1), B (2), C (3), D (4), E (5). (**b**) Mooney plots for the same rubbers. The fitted parameters are given in Table 3.

The proposed model has been used to characterize the behavior on dry samples of Ref. [27] with different concentrations of peroxide and, hence, different resulting degrees of cross-linking. Figure 6 shows the predictions of the model of the experimental points of both representations of the uniaxial test, the classical P−λ representation (Figure 6a) and the Mooney space representation (Figure 6b). The parameters obtained mainly from the Mooney plot are given in Table 3.

**Figure 7:**
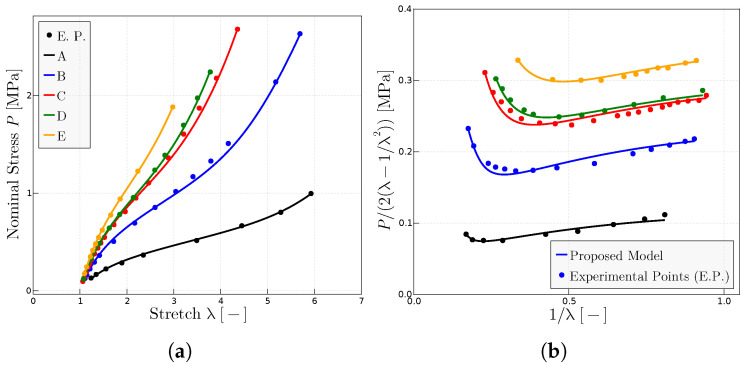
Predictions for the experimental data of rubber with different vulcanization times as given in Ref. [27]: A (t = 10 min), B (t = 40 min), C (t = 60 min), D (t = 80 min), E (t = 160 min). (**a**) Classical P−λ representation. (**b**) Mooney representation for the same rubbers. The fitted model parameters are given in Table 4.

The model has also been used to characterize the behavior of dry samples undergoing several periods of vulcanization. In this case, the tendency of the parameters is similar to what has been observed for samples with different concentrations of peroxide: for example, the parameter $P_{0}$ tends to grow while the parameter $\lambda_{u}^{\mathrm{lock}}$ tends to decrease. Nevertheless, the parameter μ grows for some rubbers while it decreases for others. Figure 7 shows how the proposed model adjusts to the experimental points both for the P−λ (Figure 7a) and Mooney (Figure 7b) representations. The parameters are given in Table 4.

**Figure 8:**
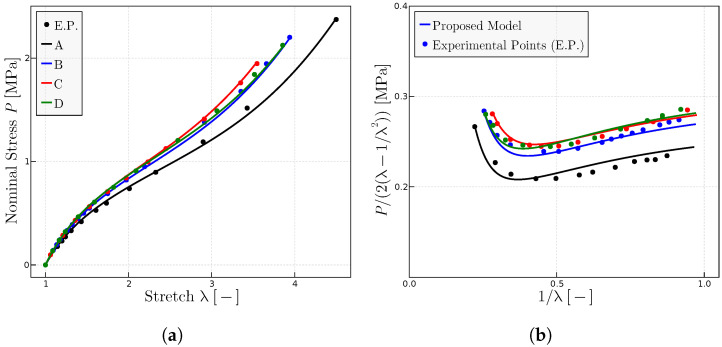
Predictions for the experimental data of rubber with different molecular weights given in Ref. [26]. (**a**) Uniaxial tests for rubbers with different initial molecular weight; values of ${\bar{M}}_{n}^{-1}$ × $10^{-6}$ of A (3.10), B (3.95), C (5.12), D (7.05). (**b**) Mooney representation for the same rubbers. The fitted parameters are given in Table 5.

Finally, the proposed model has been used for predicting the behavior of dry rubbers with different initial molecular weights; in general, the mechanical properties of the polymer increase slightly with the increase of the molecular weight. Figure 8 shows how the proposed model predicts the experimental points both in the P−λ representation (Figure 8a) and the the Mooney representation (Figure 8b). Parameters are summarized in Table 5.

### 5.4. Morris’ Experiments on Rubbers with Different Concentration of Perioxide [26]

This subsection shows the predictions of the model for a variety of rubbery materials with different concentrations of peroxide tested by Morris and reported in Ref. [26]. In Ref. [26], the author described the conditions under which the experiments have been performed.

**Figure 9:**
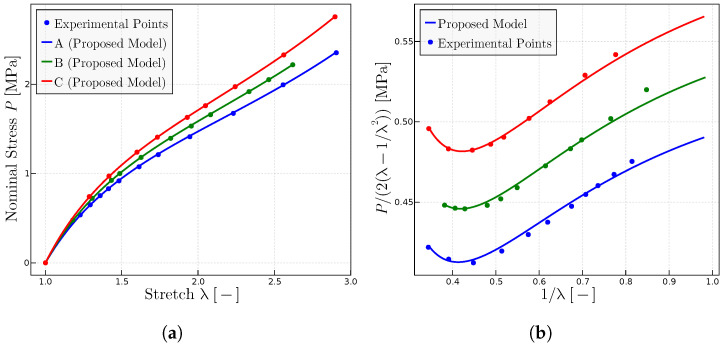
Model predictions for experiments on rubbers with different concentration of perioxide; experimental data from [26]; 2 parts dicumyl peroxide per 100 parts rubber (A, B, C). (**a**) P−λ representation. (**b**) Mooney representation for the same rubbers. The fitted parameters are given in Table 6.

**Figure 10:**
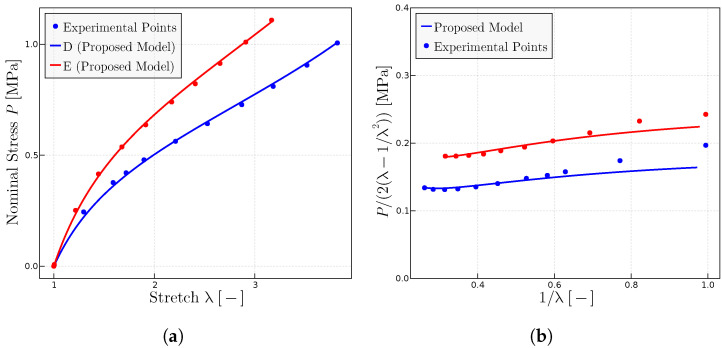
Experimental points extracted from ref. [26]. (**a**) Uniaxial tests for rubbers with different concentration of peroxide in each rubber; 1 part dicumyl peroxide per 100 parts rubber (D, E). (**b**) Mooney plots for the same rubbers. The parameters employed are given in Table 6.

Figure 9 and Figure 10 show the experimental results and their predictions for rubber at $T=25^{\circ }$ . The difference between the experimental results in both figures is the amount the dicumyl peroxide that contain the samples. Figure 9 samples have 1 part of dicumyl peroxide per 100 parts of rubber, whereas Figure 10 samples have 2 parts of dicumul peroxide per 100 parts rubber. The fitted model parameters for all experiments are given in Table 6. Figure 9b and Figure 10b plot the original Mooney space in which experimental data are given in Ref. [26]. On the contrary, Figure 9a and Figure 10a are the P−λ representations which have been obtained from the Mooney space plots.

In Ref. [26], Morris indicated, on one side, that the initial molecular weight has little effect on the minimum of the Mooney curves, while on the other side, the author indicated that the increase in the degree of the vulcanization causes changes in the minimum of the same curves. Mullins [27] obtained similar conclusions in his experiments.

On the other hand, if observing the fitted model parameters for both the upper curves (A, B, C) (Figure 9) and the lower curves (D, E) (Figure 10), they are inside of the same range (Table 6).

### 5.5. Predictions of the Kawabata et al. Experiments [55]

In this subsection, the model is used to predict the general biaxial experiments from Kawabata et al. [55]. These biaxial tests are the most general experiments in an incompressible isotropic material, because due to incompressibility, only two stretches are independent—say $\lambda_{1}$ and $\lambda_{2}$ —and only two stress values are given by the constitutive relation—say $P_{1}\left(\lambda_{1},\lambda_{2}\right)$ and $P_{2}\left(\lambda_{1},\lambda_{2}\right)$ ; the third stretch is given by the incompressibility constraint as $\lambda_{3}=1/\left(\lambda_{1}\lambda_{2}\right)$ and the pressure is given by external equilibrium, resulting in $P_{3}=0$ for plane tests. Hence, capturing both longitudinal and transverse stresses for all $\lambda_{1}-\lambda_{2}$ stretch combinations means that the general 3D behavior of the material has been captured. Whereas, to some extent, this has been achieved by other models, the callibration of the parameters for those models has been performed with several tests, so in essence, they just result in sophisticated interpolation schemes. In the present proposal, only a tensile test is used—Mooney and P−λ representations—to obtain the three parameters of the model (as one should expect in an isotropic, incompressible material) and, thereafter, predict all the test curves, both longitudinal and transverse.

**Figure 11:**
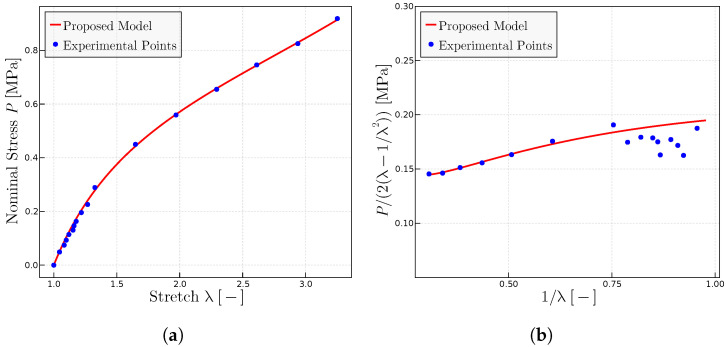

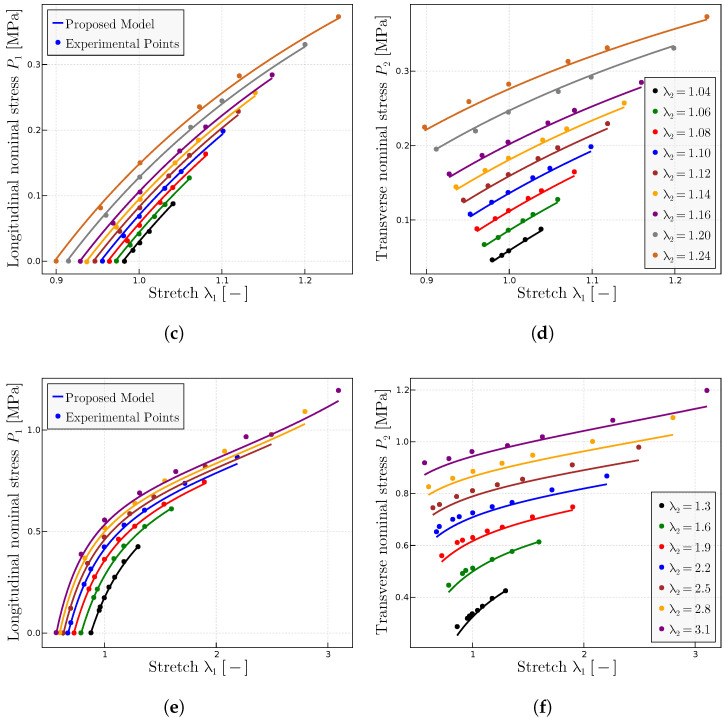
Prediction of the Kawabata et al. [55] biaxial experiments. (**a**) Extracted P−λ curve and predictions using the fitted parameters $P_{0}=1.35$ MPa, μ=0.225 MPa and $\lambda_{u}^{\mathrm{lock}}=8.0$ —for this stretches level, $\lambda_{u}^{\mathrm{lock}}$ does not affect the results in a relevant manner. (**b**) Mooney space and prediction using the same parameters. (**c**) Longitudinal nominal stresses $P_{1}\left(\lambda_{1},\lambda_{2}\right)$ and (**d**) transverse nominal stresses $P_{2}\left(\lambda_{1},\lambda_{2}\right)$ as a function of the longitudinal stretch $\lambda_{1}$ for a range of fixed small transverse stretches $\lambda_{2}$ (1.04 to 1.24). (**e**) Longitudinal nominal stresses $P_{1}\left(\lambda_{1},\lambda_{2}\right)$ and (**f**) transverse nominal stresses $P_{2}\left(\lambda_{1},\lambda_{2}\right)$ as a function of the longitudinal stretch $\lambda_{1}$ for a range of fixed large transverse stretches $\lambda_{2}$ (1.3 to 3.1). All predictions are obtained with the same material parameters of $P_{0}=1.35$ MPa, μ=0.225 MPa and $\lambda_{u}^{\mathrm{lock}}=8.0$ .

The biaxial Kawabata et al. experiments contain the tensile test as a particular case. Then, biaxial tests are used herein to extract the experimental points of the uniaxial tests [9], although we note that since the Kawabata et al. material is the same as the Treloar material, the same experimental data and the material parameters of Section 5.1 could have been used. Figure 11a,b contain the experimental points of the Kawabata et al. tensile test in classical P−λ form and in Mooney form. The best fitted parameters for this case are $P_{0}=1.35$ MPa, μ=0.225 MPa and $\lambda_{u}^{\mathrm{lock}}=8.0$ . Note that the initial points in Mooney form, whereas abundant, seem to have some relevant errors, as usual at the initial loading stages, so these points have been neglected in the fitting. The above material parameters have been kept for the rest of the predictions given in Figure 11c–f.

The predictions given in Figure 11c–f indicate that the proposed model reproduces with good accuracy the behavior of the material under general deformation patterns for a wide range and combinations of values of $\lambda_{1}$ and $\lambda_{2}$ .

### 5.6. Predictions of the Kawamura et al. Experiments in Two Silicones [56]

**Figure 12:**
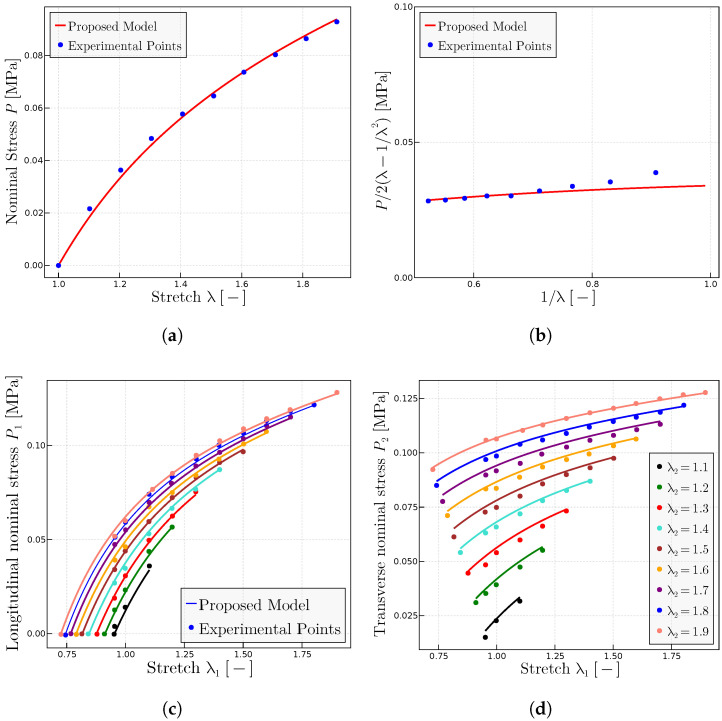
Predictions for the Kawamura et al. [56] biaxial experiments on 70% weight solution. (**a**) Extracted uniaxial test in P−λ form and prediction using the fitted parameters $P_{0}=0.235$ MPa, μ=0.0395 MPa and $\lambda_{u}^{\mathrm{lock}}=8.8$ —note that for this level of stretches, the value of $\lambda_{u}^{\mathrm{lock}}$ is not relevant as long as it sufficiently large. (**b**) Mooney space representation of the tensile test and predictions. (**c**) Longitudinal nominal stresses $P_{1}\left(\lambda_{1},\lambda_{2}\right)$ and (**d**) transverse nominal stresses $P_{2}\left(\lambda_{1},\lambda_{2}\right)$ as a function of the longitudinal stretch $\lambda_{1}$ for a range of fixed values of the transverse stretch $\lambda_{2}$ . All predictions have been obtained with the same set of material parameters, i.e., $P_{0}=0.235$ MPa and μ=0.0395 MPa.

**Figure 13:**
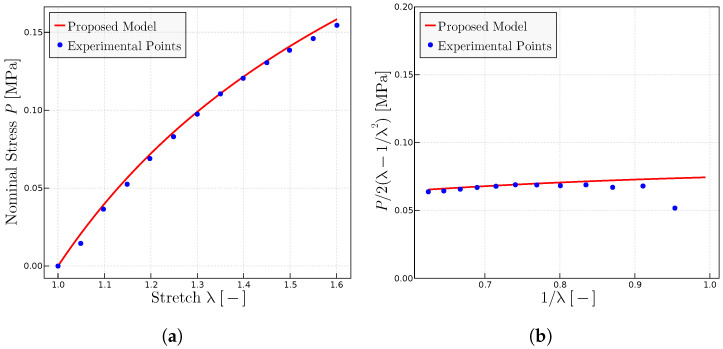

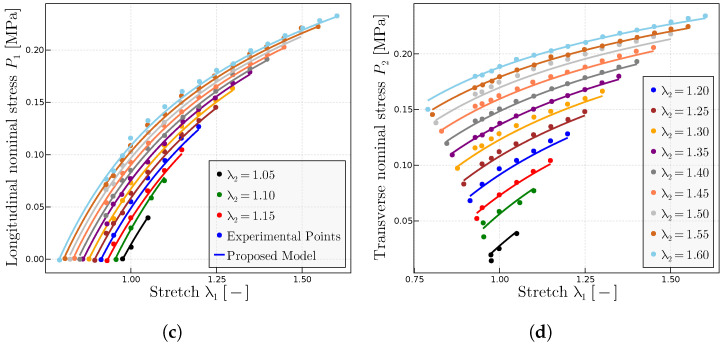
Predictions for the Kawamura et al. [56] biaxial experiments on melt solution. (**a**) Extracted uniaxial test in P−λ form and prediction using the fitted parameters $P_{0}=0.63$ MPa, μ=0.058 MPa and $\lambda_{u}^{\mathrm{lock}}=7$ —note that for this level of stretches, the value of $\lambda_{u}^{\mathrm{lock}}$ is not relevant as long as it sufficiently large. (**b**) Mooney space representation of the tensile test and predictions. (**c**) Longitudinal nominal stresses $P_{1}\left(\lambda_{1},\lambda_{2}\right)$ and (**d**) transverse nominal stresses $P_{2}\left(\lambda_{1},\lambda_{2}\right)$ as a function of the longitudinal stretch $\lambda_{1}$ for a range of fixed values of the transverse stretch $\lambda_{2}$ . All predictions have been obtained with the same set of material parameters, i.e., $P_{0}=0.63$ MPa and μ=0.058 MPa.

Kawamura [56] also performed general biaxial tests on two types of silicones, one melt silocone and one silicone in 70% weight solution. Since these are also general tests on the material, the particular case of a uniaxial test may be extracted and placed in Mooney form. The Mooney and the P−λ forms of the extracted tensile test, along their predictions, are given in Figure 12a,b, for the 70% solution material and in Figure 13a,b for the melt material. In the Mooney representation, the first points for small stretches have been discarded again because of the lack of experimental accuracy at those stretch levels. The fitted material parameters are given in the caption of the figures. It is observed in the figures that the model accurately predicts the behavior under all loading conditions using the same parameters fitted by the tensile test.

## 6. Conclusions

This work presents important insights into a recently proposed full network micro-mechanical model which employs a new micro–macro chain stretch connection. The following conclusions have been obtained:

- It is well known that the Neo-Hookean model from the classical statistical theory fails to properly represent the slope in the Mooney plots. This has been the origin of the use of the second invariant in the stored energy dependencies and the origin of the need for several additional tests to characterize such new dependence. Remarkably, Mooney plots are just a different way of plotting the tensile test data that emphasizes the small stretches range, which is important in the characterization of hyperelastic materials.
- The Neo-Hookean model is the simplest model using the chain stretch obtained from the Cauchy–Green deformation tensor, which is consistent with the affine orientation assumption of the chains used in most models. Using the same simplest Neo-Hookean chain behavior, but employing instead a chain stretch from the stretch tensor, the slope in the Mooney plots is reproduced from the same experimental data and full integration structure as in the Neo-Hookean model.
- It is well known that at small stretches, the internal energy in elastomers is relevant compared to the entropic contribution. Then, internal energy terms are also important in correctly capturing the Mooney plot slopes. The proposed model includes a term to account for that effect.
- As in the Neo-Hookean model, the proposed model may be analytically integrated in the Gaussian domain; the expression is given herein for the first time. Furthermore, it is demonstrated that the constants may be obtained directly from the Mooney plot (y-intercept and slope) or from the Mooney–Rivlin constants $C_{1}$ and $C_{2}$ .
- With the previous material parameters, obtained only from tensile test data, the model is capable of reproducing with good accuracy biaxial tests under different principal stretch ratios in the Gaussian zone. These tests represent any general loading case for isotropic incompressible hyperelastic materials. To the authors’ knowledge, the proposed model is the first analytical model capable of reproducing these general tests, including both transverse and longitudinal axes, using only two parameters obtained from a tensile test. The observed errors are smaller than those reported in model comparisons even when parameters in those works are obtained, fitting all tests simultaneously; cf. [14,22,57].
- The model accounts also for the non-Gaussian stretch domains, where locking effects are relevant. These effects produce a reorientation of the chains toward a more affine configuration. This reorientation is considered through a non-affine chain stretch. With this modification, the model captures also the different locking behaviors observed experimentally for different tests.

## Figures and Tables

**Table 1:** Parameters the proposed model used in Figure 4 for each rubber from [28].

Rubber	A	B	C	D	E	F	G
P_0_ MPa	1.925	1.925	1.925	1.925	1.925	1.925	1.925
μ MPa	0.115	0.069	0.185	0.263	0.275	0.485	0.6125
$\lambda_{u}^{\mathrm{lock}}$	7.5	7.5	7.5	7.5	7.5	7.5	7.5

**Table 2:** Parameters of the proposed model used in Figure 5 for each rubber from Ref. [28].

Rubber	A	B	C	D	E	F	G
P_0_ MPa	1.925	1.71	2.28	2.65	2.735	3.735	4.315
μ MPa	0.115	0.115	0.115	0.115	0.115	0.115	0.115
$\lambda_{u}^{\mathrm{lock}}$	7.5	7.5	7.5	7.5	7.5	7.5	7.5

**Table 3:** Fitted parameters of the model for the predictions given in Figure 6 for each rubber tested in Ref. [27]. The symbols (□, △, •, ◯, ×) are the labels given for the experimental points in Ref. [27].

Rubber	A (□)	B (▵)	C (•)	D (◯)	E (×)
P_0_ MPa	0.8	1.25	2.00	2.25	2.5
μ MPa	0.12	0.2	0.27	0.28	0.325
$\lambda_{u}^{\mathrm{lock}}$	9.7	8.25	5.6	5.25	4.3

**Table 4:** Parameters of the proposed model for the predictions given in Figure 7. The symbols correspond to the symbols used in experimental points of Ref. [27].

Rubber	A (□)	B (▵)	C (•)	D (◯)	E (×)
P_0_ MPa	0.7	1.3	1.5	1.6	2.2
μ MPa	0.14	0.3	0.4	0.38	0.35
$\lambda_{u}^{\mathrm{lock}}$	10.25	8.25	6.575	5.95	5.05

**Table 5:** Parameters of the proposed model for the predictions given in Figure 8. The symbols are the representation of the experimental points of the paper [26].

Rubber	A (•)	B (▵)	C (◯)	D (×)
P_0_ MPa	1.25	1.45	1.5	1.5
μ MPa	0.375	0.39	0.4	0.415
$\lambda_{u}^{\mathrm{lock}}$	7.0	6.4	6.1	6.65

**Table 6:** Parameters of the proposed model used in Figure 9 and Figure 10 for each rubber from [26]. The symbols are the respective representation of the experimental points in Ref. [26].

Rubber	A (▵)	B (◯)	C (□)	D (▵)	E (◯)
P_0_ MPa	4.1	4.5	4.55	0.75	1.1
μ MPa	0.35	0.35	0.4425	0.28	0.365
$\lambda_{u}^{\mathrm{lock}}$	5.15	5.0	5.175	8.75	8.75

## Data Availability

All data included in this study are available upon request by contacting the corresponding author.
